# Advanced atomic force microscopy techniques

**DOI:** 10.3762/bjnano.3.99

**Published:** 2012-12-21

**Authors:** Thilo Glatzel, Hendrik Hölscher, Thomas Schimmel, Mehmet Z Baykara, Udo D Schwarz, Ricardo Garcia

**Affiliations:** 1 Department of Physics, University of Basel, Klingelbergstr. 82, 4056 Basel, Switzerland; 2 Institute of Microstructure Technology (IMT), Karlsruhe Institute of Technology (KIT), Campus North, Hermann-von-Helmholtz-Platz 1, 76344 Eggenstein Leopoldshafen, Germany; 3 Institute of Nanotechnology (INT), Karlsruhe Institute of Technology (KIT), 76021 Karlsruhe, Germany; 4 Department of Mechanical Engineering, Bilkent University, Ankara 06800, Turkey; 5 Department of Mechanical Engineering and Materials Science, Yale University, P.O. Box 208284, New Haven, CT 06520-8284, USA; 6 Instituto de Microelectronica de Madrid, CSIC Isaac Newton 8, 28760 Tres Cantos, Madrid, Spain

**Keywords:** atomic force microscopy

Although its conceptual approach is as simple as the technique used in record players already introduced in the 19th century, the invention of the atomic force microscope (AFM) in 1986 by Binnig, Quate, and Gerber was a milestone for nanotechnology. The scanning tunneling microscope (STM), introduced some years earlier, had already achieved atomic resolution, but is limited to conductive surfaces. Since its operational principle is based on the detection of the forces acting between tip and sample, this restriction does not exist for the AFM. Consequently, atomic force microscopy quickly became the standard tool for nanometer-scale imaging of all types of surfaces in all environments. True atomic resolution was first achieved in the 1990s. The most convincing results, however, were restricted to the so-called noncontact mode in vacuum for a long time, but recent technical developments overcame this limitation, and atomic-resolution imaging is now also a standard in liquids.

Beyond pushing the resolution limit to the picometer range, the invention of the AFM triggered the development of a growing number of new scanning probe methods and approaches, ranging from an expansion of the properties that can be mapped to the active manipulation of surfaces and small particles. Practically every month, reports on the growing capabilities of AFMs appear. Nearly every physical effect that influences the tip–sample interaction has been used to improve existing modes and to develop new ones. For example, many recently presented techniques include the excitation of higher cantilever oscillation modes; it is amazing in how many ways the shaking of a simple cantilever can improve our knowledge about the tip–sample interaction. Another direction is high-speed atomic force microscopy, which is one of the eminent challenges that need to be solved in order to allow the in situ observation of biological processes. Data acquisition times have already reached the millisecond range, enabling the visualization of the dynamic behavior of biological molecules and cells. Other recent accomplishments include imaging of organic molecules with unprecedented resolution, full three-dimensional mapping of surface force fields, and the imaging and discrimination of individual chemical bonds.

The development of advanced techniques is the focus of this Thematic Series, following the Thematic Series “Scanning probe microscopy and related techniques” edited by Ernst Meyer and the Thematic Series “Noncontact atomic force microscopy” edited by Udo Schwarz. The articles that are part of the series demonstrate that, despite its 25 years of history, the AFM is still far from reaching its limits, and today’s developments are far-reaching. As the number of research groups utilizing advanced atomic force microscopy techniques increases with each passing year, the technical improvements, data-acquisition approaches, analysis procedures, user friendliness, and application areas of the technique further diversify. With this Thematic Series, it is our intention to stimulate these improvements.

We thank all authors for contributing their excellent work to this series. Furthermore, we acknowledge all referees for their promptly provided reports keeping the publication times short and attractive for contributors. Finally, we are grateful to the open access policy of the *Beilstein Journal of Nanotechnology* providing the ground for unrestricted discussions on advanced atomic force microscopy techniques.

Thilo Glatzel, Hendrik Hölscher, Thomas Schimmel, Mehmet Z. Baykara, Udo D. Schwarz and Ricardo Garcia

December 2012

